# Application of oxygen saturation variability analysis for the detection of exacerbation in individuals with COPD: A proof‐of‐concept study

**DOI:** 10.14814/phy2.15132

**Published:** 2021-12-01

**Authors:** Ahmed Al Rajeh, Amar S. Bhogal, Yunkai Zhang, Joseph T. Costello, John R Hurst, Ali R. Mani

**Affiliations:** ^1^ UCL Respiratory Royal Free Campus Division of Medicine University College London London UK; ^2^ Department of Respiratory Care King Faisal University Al‐Ahsa Saudi Arabia; ^3^ Network Physiology Laboratory Division of Medicine UCL London UK; ^4^ Medical School University of Birmingham Birmingham UK; ^5^ Extreme Environment Laboratory School of Sport, Health and Exercise Science University of Portsmouth Portsmouth UK

**Keywords:** entropy, physiological measurement, Pulse Oximetry, respiratory, S_p_O_2_

## Abstract

**Background:**

Individuals with chronic obstructive pulmonary disease (COPD) commonly experience exacerbations, which may require hospital admission. Early detection of exacerbations, and therefore early treatment, could be crucial in preventing admission and improving outcomes. Our previous research has demonstrated that the pattern analysis of peripheral oxygen saturation (S_p_O_2_) fluctuations provides novel insights into the engagement of the respiratory control system in response to physiological stress (hypoxia). Therefore, this pilot study tested the hypothesis that the pattern of S_p_O_2_ variations in overnight recordings of individuals with COPD would distinguish between stable and exacerbation phases of the disease.

**Methods:**

Overnight pulse oximetry data from 11 individuals with COPD, who exhibited exacerbation after a period of stable disease, were examined. Stable phase recordings were conducted overnight and one night prior to exacerbation recordings were also analyzed. Pattern analysis of S_p_O_2_ variations was carried examined using sample entropy (for assessment of irregularity), the multiscale entropy (complexity), and detrended fluctuation analysis (self‐similarity).

**Results:**

S_p_O_2_ variations displayed a complex pattern in both stable and exacerbation phases of COPD. During an exacerbation, S_p_O_2_ entropy increased (*p *= 0.029) and long‐term fractal‐like exponent (α2) decreased (*p* = 0.002) while the mean and standard deviation of S_p_O_2_ time series remained unchanged. Through ROC analyses, S_p_O_2_ entropy and α2 were both able to classify the COPD phases into either stable or exacerbation phase. With the best positive predictor value (PPV) for sample entropy (PPV = 70%) and a cut‐off value of 0.454. While the best negative predictor value (NPV) was α2 (NPV = 78%) with a cut‐off value of 1.00.

**Conclusion:**

Alterations in S_p_O_2_ entropy and the fractal‐like exponent have the potential to detect exacerbations in COPD. Further research is warranted to examine if S_p_O_2_ variability analysis could be used as a novel objective method of detecting exacerbations.

## Graphical abstract

This report provides evidence that the pattern of peripheral oxygen saturation (S_p_O_2_) fluctuations detects exacerbations in individuals with COPD. The entropy of S_p_O_2_ signal increases a day prior to clinical diagnosis of exacerbation in COPD while mean and total variability of S_p_O_2_ signals remain unchanged. This finding has potential for development of a non‐invasive method for early detection of exacerbations in COPD.

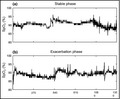

**New & Noteworthy**

This report provides evidence that the pattern of peripheral oxygen saturation (S_p_O_2_) fluctuations detects exacerbations in individuals with COPD. The entropy of S_p_O_2_ signal increases a day prior to clinical diagnosis of exacerbation in COPD while mean and total variability of S_p_O_2_ signals remain unchanged. This finding has the potential for development of a non‐invasive method for early detection of exacerbations in COPD.

## 1 INTRODUCTION

Chronic obstructive pulmonary disease (COPD) is a global health burden estimated to affect 251 million people worldwide and carries with it high mortality (Husebø et al., 2014; Mathers & Loncar, 2006). In COPD, individuals commonly experience exacerbations of their illness leading to a sudden deterioration in their health (Al Rajeh et al., 2020). Patients report that exacerbations are the most disruptive aspect of living with COPD (Zhang et al., 2018). This often leads to hospital admissions; with poor prognosis (Al Rajeh et al., 2020). Consequently, the prevention of exacerbations is essential when managing COPD (Hurst, Vestbo, et al., 2010) and better prevention of COPD exacerbations has been identified as a top research priority (Alqahtani et al., 2021).

Although several tools have been proposed to help detect exacerbations earlier, to the best of our knowledge, there is no sensitive method of predicting exacerbation risk or rate accurately (Adibi et al., 2020; Al Rajeh et al., 2020; Donaldson et al., 2012; Hurst, Vestbo, et al., 2010). Clinical studies have, however, attempted to investigate the utility of changes in various physiological parameters including heart rate, forced expiratory volume (FEV1), respiratory rate and level of peripheral oxygen saturation (S_p_O_2_) before and during exacerbation with limited success (Al Rajeh et al., 2020.; Al Rajeh & Hurst, 2016; Burton et al., 2015; Hurst, Donaldson, et al., 2010; Hurst, Vestbo, et al., 2010; Husebø et al., 2014). While pulse oximetry has some role when monitoring individuals in the community, there is no clear benefit of using mean values and overnight readings with the prediction of exacerbations due to the insignificant magnitude of the changes (Al Rajeh et al., 2020).

Recent evidence has demonstrated that the complex pattern of variability in S_p_O_2_ signals may provide more mechanistic insight than absolute or mean S_p_O_2_ values (Bhogal & Mani, 2017; Costello et al., 2020; Jiang et al., 2021). S_p_O_2_ signals exhibit a complex fractal‐like pattern in hypoxic individuals (Costello et al., 2020). Using a network physiology approach, we have demonstrated that S_p_O_2_ fluctuations are not random and contain useful information about the engagement of respiratory control during hypoxia (Jiang et al., 2021). Furthermore, we also reported that S_p_O_2_ entropy, but not absolute or mean S_p_O_2_, was correlated with the perception of breathlessness in the same, otherwise healthy, individuals when hypoxic (Costello et al., 2020). These data suggest that there is a significant exchange of information between S_p_O_2_ and other respiratory variables (i.e. tidal volume, minute ventilation, respiratory rate, end‐tidal oxygen, and carbon dioxide pressure) during graded normobaric hypoxia in healthy participants (Jiang et al., 2021). Fluctuations in these respiratory variables were reflected in the S_p_O_2_ signal, specifically in S_p_O_2_ entropy (Jiang et al., 2021), a measure that describes the unpredictability and irregularity of these S_p_O_2_ signals (Richman & Moorman, 2000). Calculated using a well‐established algorithm (Richman & Moorman, 2000), S_p_O_2_ entropy may reveal additional information about cardiorespiratory control in health and disease (Jiang et al., 2021).

To date, the usefulness of S_p_O_2_ variability analysis has not been studied extensively in COPD. Accordingly, this pilot study investigated the hypothesis that S_p_O_2_ variability would distinguish between the two phases of COPD (stable vs. exacerbation). As S_p_O_2_ entropy is easily computed and incorporated into bedside monitors or smart devices, this method could assist in the earlier detection of COPD exacerbations and, following faster access to the necessary treatment, ultimately result in an improved prognosis (Qureshi et al., 2014; Wilkinson et al., 2004).

## 2 METHODS

### 2.1 Participants

From September 2016 to January 2018, participants were recruited from COPD clinics and pulmonary rehabilitation classes at three separate sites in London. All participants were fully informed and submitted written consent forms. The UK Health Research Authority and Royal Free Hospital local committee granted ethical approvals on data collection (16/LO/1120). The inclusion criteria consisted of COPD diagnosis [smoking history ≥10 pack years and post‐bronchodilator FEV1/FVC <0.7 (suggesting a non‐reversible obstructive lung disease pattern)], one or more self‐reported moderate or severe exacerbations of their COPD in the last 12 months, and the ability to attend scheduled appointments and use study equipment. Individuals were excluded if they had an existing diagnosis of obstructive sleep apnea either via self‐report or results of STOP‐Bang and Epworth questionnaires (Johns, 1991; Nagappa et al., 2015), and/or significant co‐morbidities that prevented participation (Al Rajeh et al., 2020).

The clinical recordings used for analysis are credited to a recently published pilot randomized controlled trial regarding COPD exacerbation detection (Al Rajeh et al., 2020). The data in this analysis derives from one arm of this study looking at overnight monitoring of COPD (n = 44). Some of the data, including individual demographics and mean S_p_O_2_, but importantly not any S_p_O_2_ variability data, have already been published in the referenced study (Al Rajeh et al., 2020). In the original study, only 13 participants exacerbated in the time frame of the study and were included in the analysis. The quality of S_p_O_2_ recording for two individuals was limited (less than 90 mins continuous S_p_O_2_ signal) and therefore these participants were excluded from the analysis (n = 11). In the study, there were 7 male participants and 4 female participants (n = 11) with an average age (SD) of 71.8 (10.4) years. Of these participants, 3 were current smokers, with 8 ex‐smokers. The baseline clinical characteristics for the population studied can be found in Table 1.

**TABLE 1:** Summary of the baseline demographics of the study participants

	Age	BMI	MRC Dyspnea Scale	FEV1 (%)
All Participants (n = 11)	71.8 ± 10.4	24.6 ± 6.70	2.82 ± 0.874	47.7 ± 18.8

All data are expressed as mean ±SD

### 2.2 Data collection

Each participant received a wristband pulse oximeter (Nonin 3150, Nonin Medical Inc.) and was instructed by researchers on using it. Each pulse oximeter measured the S_p_O_2_ time series of each participant overnight. In the original clinical trial, participants were closely monitored during the first 2 weeks of the study to ensure they were using the equipment properly and recording data accurately. They were considered “stable” during this 2‐week period when there was no incidence of exacerbation. An exacerbation was defined as the need for oral corticosteroids or antibiotics, as judged by the patient's clinician or self‐management plan (Al Rajeh et al., 2020). The first set of continuous S_p_O_2_ time series available from the stable phase was used for the calculation of S_p_O_2_ variability indices in this study. The sampling rate of S_p_O_2_ recording was 0.25 Hz (one sample recorded every 4 s). S_p_O_2_ signals were saved as comma‐separated value (csv.) files for stability and one day prior to exacerbation phase separately. The data were chosen one day prior to exacerbation, as this offered insight into a useful window for the early detection of an exacerbation. In this report, the signals recorded a day prior to clinical diagnosis of exacerbation are called “exacerbation phase”.

### 2.3 S_p_O_2_ variability

The longest duration of time that all individuals had of uninterrupted S_p_O_2_ data was ~90 min, so the first available 90‐min recording was used for the analyses. Well‐established measures within this field to analyze the patterns of variability (Bhogal & Mani, 2017), including standard deviation (SD), sample entropy, Multiscale Entropy (MSE), and Detrended Fluctuation Analysis (DFA) (Bhogal & Mani, 2017) were employed.

Details of these methods and associated algorithms are described in detail elsewhere [DFA (Peng et al., 1995), MSE (Costa et al., 2002), and sample entropy (Richman & Moorman, 2000)]. In brief, sample entropy looks at the complexity of a time series by analyzing the probability of repetition of a signal, with a particular length (*m*) and degree of tolerance (*r*). In this study, sample entropy was determined under the settings of *m* at 2 and *r* at 0.2 as previously described (Richman & Moorman, 2000). MSE looks at entropy at different time scales, and as such is seen as an extension of sample entropy. The trends of entropy change within this time scales provide further information on the complexity of a data set. For this analysis, MSE was used over five scales in accordance with current practice (Costa et al., 2002). Finally, to examine the fractality of the S_p_O_2_ data, we employed DFA as it looks at the self‐similarity of a time series providing information on the fractal‐like dynamics present (Peng et al., 1995). In a DFA plot, the logarithm of fluctuation (standard deviation) of detrended time series is plotted against the logarithm of scale (*n*). The slope of this line is known as the scaling exponent (α). Previous studies proved there to be a “cross‐over” in S_p_O_2_ DFA graph, thus the short‐term and long‐term scaling exponent, α1 and α2 are calculated separately as described elsewhere (Bhogal & Mani, 2017). All calculations were completed in MATLAB (Matworks R2020b).

### 2.4 Statistical analysis

All statistical tests were performed using MATLAB (Matworks R2020b) and SPSS software. A paired two‐tailed Student's t‐test was employed for comparing the mean, SD, α1 and α2 of COPD individuals (n = 11) during a stable phase to that of the same cohort a day prior to the clinical diagnosis of an exacerbation. A two‐way ANOVA was used to analyze the results of MSE, with statistical significance taken as a *p*‐value less than 0.05. Receiver operating characteristic (ROC) curves of sample entropy at 5 scales, α1 and α2 of individuals were plotted by SPSS for further investigation of the differences in these indices between the two phases and their potential to detect early exacerbation (exacerbation phase). Area under the curve (AUC), sensitivity, specificity, positive predictive value (PPV), negative predictive value (NPV), and a cut‐off value of each index were also determined from ROC curves.

## 3 RESULTS

### 3.1 Pattern analysis of S_p_O_2_ variability

**FIGURE 1:**
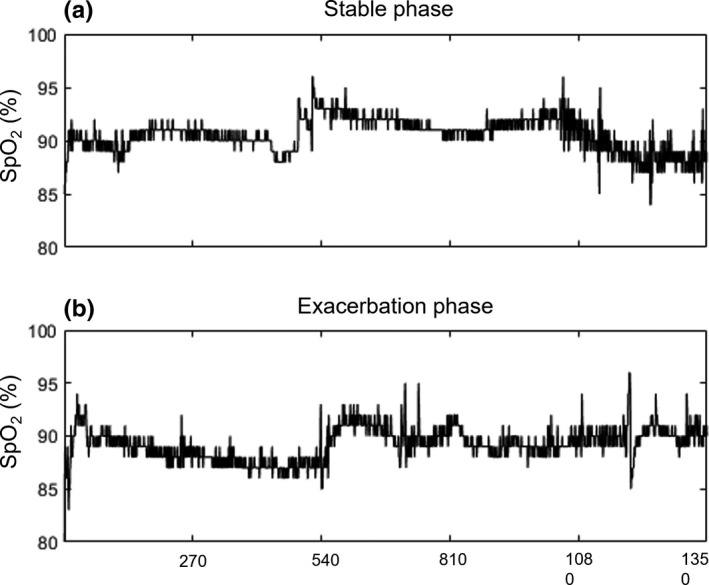
Representative 90‐minute S_p_O_2_ signals recorded from an individual with COPD at (a) stable phase and (b) a day prior to clinical diagnosis of exacerbation (exacerbation phase). X‐axis is the data points of the pulse oximeter signals recording (1 sample every 4 seconds), and Y‐axis is the S_p_O_2_ (%)

The S_p_O_2_ signals for exacerbation and stable phase show a complex pattern (see Figure 1). A summary of mean S_p_O_2_ and the various variability indices are displayed in Table 2. Overall, mean S_p_O_2_ during the stable phase was not statistically different to that of the exacerbation phase (91.4 ± 1.89% vs. 90.6 ± 2.11%; *p* = 0.125), likewise, the mean SD of both phases were similar (Table 2).

**TABLE 2:** Summary of S_p_O_2_ mean and variability indices in 11 individuals with COPD during stable phase and exacerbation phase

	Mean S_p_O_2_ (%)	Standard deviation	Sample entropy	DFA (α1)	DFA (α2)
COPD stable	91.4 ± 1.89	1.33 ± 0.440	0.395 ± 0.101	1.17 ± 0.110	1.04 ± 0.114
COPD exacerbation	90.6 ± 2.11	1.33 ± 0.444	0.505 ± 0.159	1.15 ± 0.137	0.925 ± 0.107
*p*‐value	0.125	0.963	**0.029**	0.555	**0.002**

All data are expressed as mean ±SD, and the p‐value is calculated using a Student's paired t‐test.

Bold values reflect a statistically significant difference between the groups (*p*‐value < 0.05).

**FIGURE 2:**
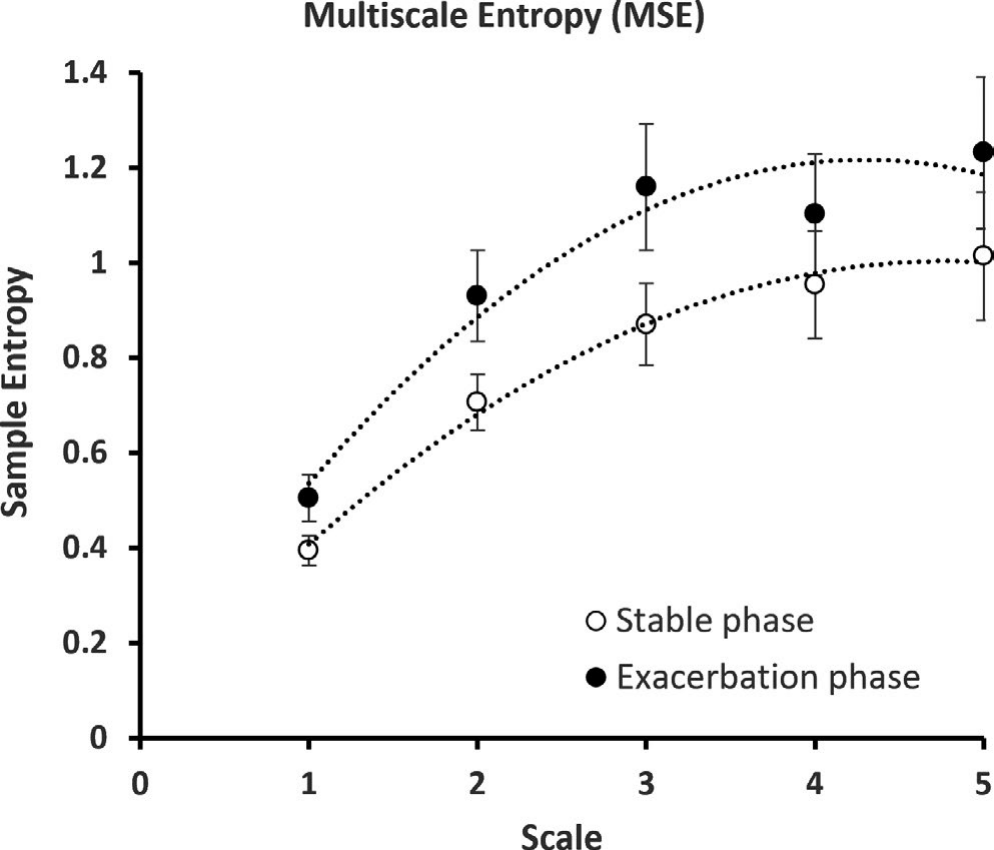
Multiscale entropy (MSE) graph describing the overall complexity of the individuals with COPD at stable phase and exacerbation. The error bars are calculated sample error of the mean values

Mean sample entropy increased (0.395 ± 0.101 vs. 0.505 ± 0.159; *p *< 0.05) during exacerbation. This indicates an increased irregularity of the signal; however, in order to assess whether this change was random or complex, we analyzed the data using MSE (Bhogal & Mani, 2017). This difference is constantly observed across the increasing scale factor using MSE (Figure 2), where the values of mean sample entropy during exacerbation were all higher than that of the stable phase. Two‐way ANOVA analysis showed that there was a significant difference in the MSE between the stable phase and exacerbation phase (F_group_ = 8.63, *p* = 0.004), highlighting the increased amount of information and complexity during an exacerbation. Additionally, an increasing trend of sample entropy value from scale 1 to 5 in both COPD phases was observed (Figure 2), revealing that fluctuated S_p_O_2_ time series is not a random process (Costa et al., 2002).

From DFA, the short‐term scaling exponent, α1 of the stable phase (α1 = 1.17 ±0.110) and exacerbation (α1 = 1.15 ± 0.137) were between values expected from Brownian noise (α = 1.50) and 1/f dynamics (α = 1.00); however, their values did not differ between phases (*p* = 0.55). However, the long‐term scaling exponent, α2, of both phases approached closer to 1/f dynamics (α2 = 1.04 ± 0.114 in stable and α2 = 0.925 ± 0.107 in exacerbation), confirming that S_p_O_2_ fluctuations have a fractal‐like pattern (Bhogal & Mani, 2017). Statistical significance was shown between α2 of exacerbation vs stable phase (*p *< 0.01), validating there was a slight reduction in scaling exponent and shift toward white noise dynamics during exacerbation. Two example graphs of the DFA analysis obtained from the S_p_O_2_ time series are shown in Appendix A.

### 3.2 ROC analysis of S_p_O_2_

**FIGURE 3:**
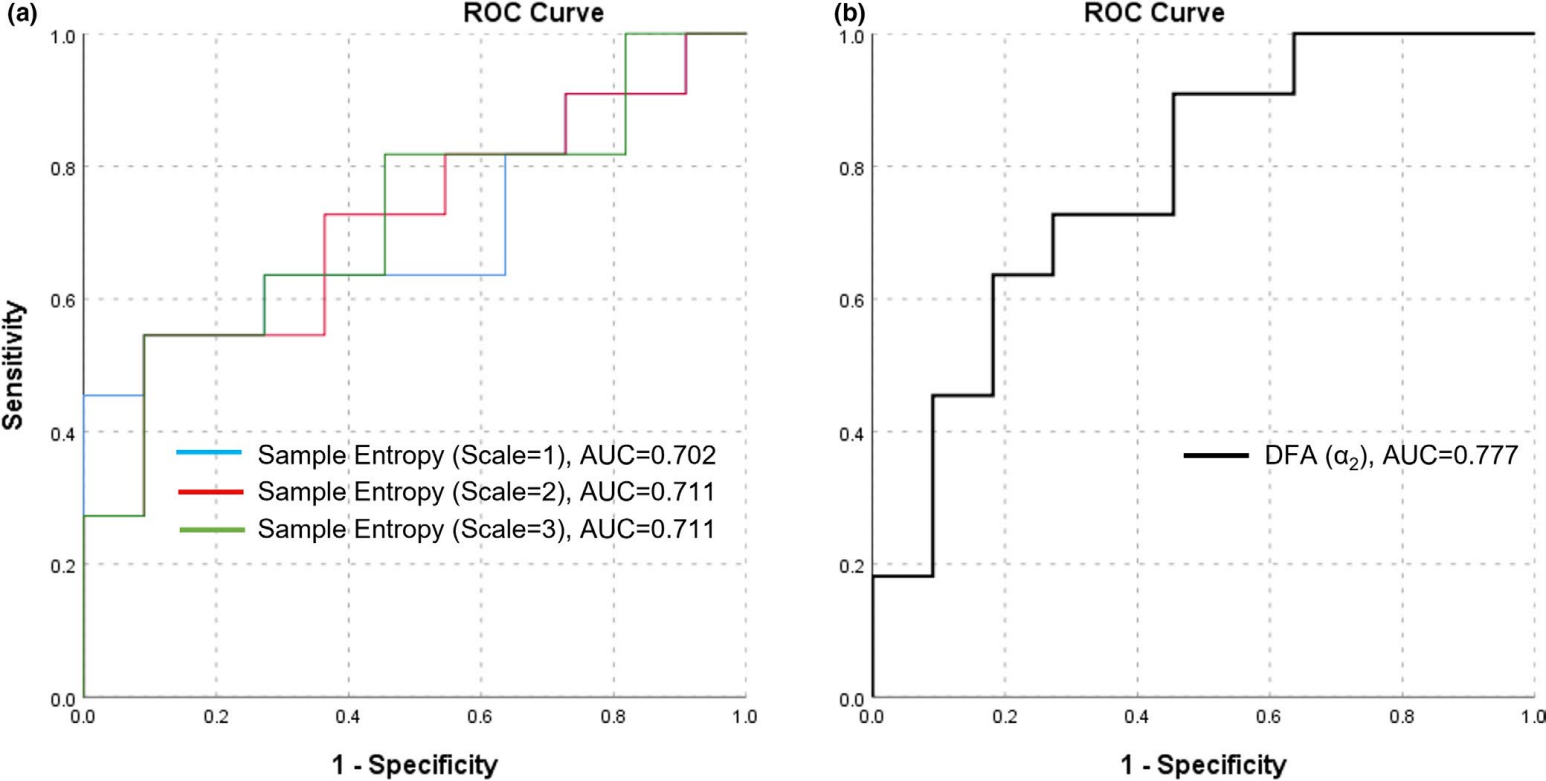
ROC curve for classifying COPD phase (stable or exacerbation) based on S_p_O_2_ variability indices

ROC curves assessing the sensitivity and specificity of S_p_O_2_ variability indices in classifying stable from exacerbation phase are presented in Figure 3. Sample Entropy and α2 exhibited a significant AUC with values of 0.702 and 0.777 respectively (Table 2). MSE indices at scales 2 and 3, also had a significant statistical AUC with values of 0.711 and 0.719 respectively (Table 2). The best positive predictor value (PPV) was for sample entropy (PPV = 70%) with a cut‐off value of 0.454. The best negative predictor value (NPV) was for α2 (NPV = 78%) with a cut‐off value of 1.00.

## 4 DISCUSSION

This study tested the hypothesis that the pattern of S_p_O_2_ variations in overnight recordings of individuals with COPD would distinguish between stable and exacerbation phases. In support of the hypothesis, our novel findings suggest that sample entropy at different scales increases, while the long‐term scaling exponent (α2) decreases, a day prior to the clinical diagnosis of an exacerbation of COPD. These indices were also different during the stable and exacerbation phases, while mean S_p_O_2_ remained stable throughout. Furthermore, in terms of sensitivity, specificity, PPV, and NPV from ROC analyses, sample entropy of the original S_p_O_2_ time series and α2 of DFA appear to have the best diagnostic capabilities to support earlier detection of COPD exacerbations.

This study extends our earlier work in S_p_O_2_ variability analysis in healthy individual after exposure to hypoxia to individuals with chronic lung disease (Bhogal & Mani, 2017; Costello et al., 2020; Jiang et al., 2021). We have shown an increased in sample entropy in healthy participants during exposure to normobaric hypoxia (Costello et al., 2020; Jiang et al., 2021) and here we have similarly demonstrated a higher sample entropy 0.395–0.505, and in all scales in MSE, following COPD exacerbation. Interestingly, these previous reports established that there is an inverse correlation between mean S_p_O_2_ and S_p_O_2_ Sample Entropy under both normoxic and hypoxic environments in healthy individuals (Bhogal & Mani, 2017; Costello et al., 2020), with lower oxygen saturation correlated with higher S_p_O_2_ entropy. This relationship was not observed in the current study (Appendix B), which could suggest a compromise in the cardiorespiratory integrity in COPD (O’Donnell et al., 2020). Another consideration for the lack of correlation between mean S_p_O_2_ and sample entropy in our cohort; could be the wide range of S_p_O_2_ values included in the present study (individuals with COPD) versus the other reports in healthy participants. Nevertheless, future studies with a larger number of participants could test this hypothesis.

According to Pincus (1994), higher entropy signifies greater amounts of information being processed in a complex physiological system, reflecting the enhanced connections and communications across various components within that system (Pincus, 1994). In terms of the cardiorespiratory system and its homeostatic control of oxygen saturation, Jiang et al. (2021) provided further insight by using a network physiology approach to show that the information controlling oxygen saturation was communicated across several key components of the cardiorespiratory system. Therefore, when this system is under hypoxic stress either through a decrease in the fraction of inspired oxygen or in a clinical state (COPD); the transfer of information is increased across these components to maintain mean S_p_O_2_. This is demonstrated by the rise in sample entropy when healthy individuals are hypoxic (Costello et al., 2020), as well as during an exacerbation in COPD (see Figure 2 and Table 2).

The sample entropy in both stable and exacerbation phases of COPD (0.395 ± 0.101 vs. 0.505 ± 0.159) is notably less than the sample entropy value of healthy individuals (0.98 ± 0.28) with the same mean value of mean S_p_O_2_ (93.94 ± 1.85%) during hypoxic challenge (Jiang et al., 2021). This may be attributed to the disruption of functional connectivity within cardiorespiratory system when COPD is diagnosed (Donaldson et al., 2012). This is reflected in the impaired response to hypoxia and changes in ventilation that often lead to hypercapnia (Abdo & Heunks, 2012). This disruption in the control system limits the adaptive response to hypoxia during exacerbation, thus reflected by a limited increase in sample entropy. This hypothesis requires further examination with more stringent control of possible confounders such as age, lifestyle (e.g., smoking), and environment. For example, we have reported that aging reduces S_p_O_2_ entropy in otherwise healthy individuals (Bhogal & Mani, 2017). This supports the theory that the integrity of cardiorespiratory control system is affected by aging and chronic diseases such as COPD (O’Donnell et al., 2020; Strait & Lakatta,). However, future studies should aim to compare S_p_O_2_ entropy between age‐matched healthy cohorts and COPD individuals in different phases to help explain the changes seen in an exacerbation and better predict future exacerbations.

It is now well‐established that the DFA of S_p_O_2_ signals results in two scaled components, one representing short‐term (α1) and the other long‐term (α2) fractal‐like fluctuations (Bhogal & Mani, 2017). Table 2 illustrates a statistically significant decrease in α2 upon exacerbation while α1 remains stable. Interestingly, this data trend contradicts a study assessing DFA’s usefulness in diagnosing childhood sleep apnea‐hypopnoea (Vaquerizo‐Villar et al., 2018). Like COPD, sleep apnea is also associated with hypoxia; however, it is due to episodic upper airway collapse during sleep (Stradling et al., 2004). By applying DFA in their study, Vaquerizo‐Villar et al. (2018) observed an increased α1 with intensified apnea‐hypopnoea severity while α2 was unaltered. These results are likely due to the different underlying pathophysiology in the two diseases. Despite both leading to dyspnea, apnea‐hypopnoea is associated with acute episodic hypoxia reflected by alternation in the short‐term scaling component (α1). While in COPD, individuals suffer from chronic hypoxia leading to changes in the long‐term scaling component (α2) (Khatri & Ioachimescu, 2016). Furthermore, the faster breathing pattern in younger children, and the associated dynamics of apnea‐hypopnoea occurrences, may also translate to shorter time scales being more sensitive than longer time scales in disease relative to adults.

Although the values of the ROC analysis of the sample entropy and α2 showed moderate levels of sensitivity and specificity, this was the first attempt to suggest their potential in supporting earlier diagnosis of COPD exacerbations. Judging from the ‘zigzag’ shape of the ROC analysis (Figure 3) and repeated values in sensitivity and specificity (Table 3), we speculate that this was due to the small sample size in the current study (n = 11) or insufficient heterogeneity/range in the predictor measures. Previous work had proposed the effectiveness of COPD Assessment Test (CAT) questionnaire to evaluate disease severity, as CAT score significantly increases during exacerbation (Mackay et al., 2012). However, this method showed an AUC of 0.64 for the CAT questionnaire in 491 COPD individuals in detecting exacerbations (Lee et al., 2014), which is less than that of both sample entropy (AUC = 0.702) and α2 (AUC = 0.777) in our analysis. CAT is a well‐established scoring system for monitoring COPD, but its diagnostic value may be compromised by its subjectivity. As S_p_O_2_ analysis is a useful predictor of dyspnea, and a key component of the CAT questionnaire (Costello et al., 2020), combining the objective S_p_O_2_ analysis with the CAT questionnaire could provide the most accurate predictor of future exacerbations.

**TABLE 3:** Summary for ROC analysis of S_p_O_2_ variability indices for detection of exacerbation

	AUC	*p*‐value	Cut‐off	Sensitivity	Specificity	PPV	NPV
Sample entropy	0.702	**0.029**	0.454	0.636	0.727	0.700	0.666
Sample entropy (scale 2)	0.711	**0.016**	0.758	0.727	0.636	0.666	0.700
Sample entropy (scale 3)	0.719	**0.031**	0.836	0.818	0.545	0.643	0.750
Sample entropy (scale 4)	0.628	0.175	0.844	0.818	0.636	0.692	0.778
Sample entropy (scale 5)	0.636	0.120	0.903	0.818	0.636	0.692	0.778
DFA (α1)	0.529	0.555	1.17	0.545	0.455	0.500	0.500
DFA (α2)	0.777	**0.002**	1.00	0.818	0.636	0.692	0.778

Abbreviations: AUC, area under the curve, PPV, positive predictive value, NPV, negative predictive value.

Bold values reflect a statistically significant difference between the groups (*p*‐value < 0.05).

In the present data, we had access to 90 min of signal in all individuals and showed that S_p_O_2_ variability indices can distinguish between stable and exacerbation phases. In a preliminary analysis, we also examined shorter intervals of S_p_O_2_ signals (i.e., 60, 50, 40, 30, and 15 min). As shown in Appendix C, we applied Bland‐Altman analysis to the data and found that 60 min is also sufficient for S_p_O_2_ sample entropy analysis, while DFA requires 90 min to detect an exacerbation. While this might not be clinically feasible for spot‐checking hypoxia in acute scenarios, this analysis combined with better S_p_O_2_ wearable technology may improve management in individuals experiencing chronic hypoxia (Buekers et al., 2019).

### 4.1 Limitations and future research

Like other pilot studies, the major limitation of the current study is the small sample size. The source of S_p_O_2_ recording data in this study was from a pilot randomized controlled trial testing the effectiveness of overnight physiological monitoring to predict COPD exacerbation (Al Rajeh et al., 2020). With the limited sample, there is a risk of low statistical power and type II error. However, despite the small sample size, our results reached statistical significance. This demonstrates the potential of S_p_O_2_ variability analysis in non‐invasively detecting early exacerbations for timely treatment and the need for future studies with larger sample sizes. This method also has the potential to monitor exacerbation recovery and provide an objective tool for discharge in these individuals, and future studies can help determine this.

Since data were obtained from a randomized clinical trial with regular follow‐up of the participants, the chance of selection bias is low. However, a possible source of bias in this study is the availability of long (>90 min) continuous S_p_O_2_ signal in the participants. In the present study, two participants had less than 90 min continuous S_p_O_2_ signals in their stable phase and were not included in this study. Future studies can investigate this limitation in a larger multicentre trial to assess the value of S_p_O_2_ pattern analysis in the prediction of exacerbation.

It is difficult to estimate an accurate cut‐off for separation of stable versus exacerbation phase based on such a small sample size. To further test the validity of the cut‐off value, we randomly selected samples from stable periods of the same participants recorded at different days and measured S_p_O_2_ Sample Entropy. The mean (±SD) of the randomly selected samples was 0.341 ± 134 (n = 11) which was not significantly different from data presented in Table 2 for the S_p_O_2_ Sample Entropy of stable phase (0.395 ± 0.101). Furthermore, the rate of false positive was 18% when the cut‐off in Table 3 was used for the prediction of exacerbation. While these pilot results are promising, a comprehensive analysis of the reportability of S_p_O_2_ variability indices is required prior to the translation of these findings into clinical practice.

Another limitation of this study is the severity of exacerbation was not measured. An exacerbation was defined as the need for oral corticosteroids or antibiotics, as judged by the patient's clinician or self‐management plan. This practical approach has its shortcomings as the prescription of oral corticosteroids/antibiotics following the worsening of respiratory symptoms may vary among practitioners and healthcare systems (Celli et al., 2021 Sep 27).

Hurst et al., previously reported that a combined oximetry score (i.e., the positive magnitude in standard deviation units of the fall in S_p_O_2_ and the rise in heart rate) could predict the onset of an exacerbation, prior to clinical diagnosis (Hurst, Donaldson, et al., 2010). We had limited access to high quality continuous signals 2–3 days prior to diagnosis of exacerbation in the current study and could only include S_p_O_2_ variability analysis one day prior to clinical diagnosis of exacerbation. Therefore, future studies can extend our pilot study by developing wearable devices suitable for long‐term signal recording for S_p_O_2_ fluctuation analysis. In addition, similar to this combined oximetry score, novel analytical methods (e.g., transfer entropy) have the potential to assess the interaction of heart rate and S_p_O_2_ time series in order to develop a comprehensive physiomarker for the non‐invasive assessment of patients with COPD. Application of these methods in healthcare warrant further investigations in larger studies.

## 5 CONCLUSION

This is a proof‐of‐concept study demonstrating that S_p_O_2_ fluctuation analysis has the potential to be used to support earlier detection of exacerbations in individuals with COPD. Specifically, the sample entropy increases and there is an alteration in fractal‐like behavior of S_p_O_2_ fluctuations during exacerbation. As pulse oximetry has recently been expanded beyond the measurement of absolute peripheral oxygen saturation, measurement of S_p_O_2_ dynamics has the potential to be incorporated into smart devices to assist the early diagnosis of COPD exacerbations.

## CONFLICT OF INTEREST

The authors declare that the research was conducted in the absence of any commercial or financial relationships that could be construed as a potential conflict of interest.

## AUTHOR CONTRIBUTIONS

Ahmed Al Rajeh and John R Hurst conceived and designed the original clinical study and collected clinical data. Amar S Bhogal, Joseph T. Costello, and Ali R Mani formulated the concept of oxygen saturation variability analysis in COPD. Yunkai Zhang and Ali R Mani performed the computational analysis and evaluated the data. Amar S Bhogal, Yunkai Zhang and Ali R Mani wrote the first manuscript draft and all authors revised it for important intellectual content. All authors read and approved the final manuscript.

## Figures and Tables

**FIGURE A1:**
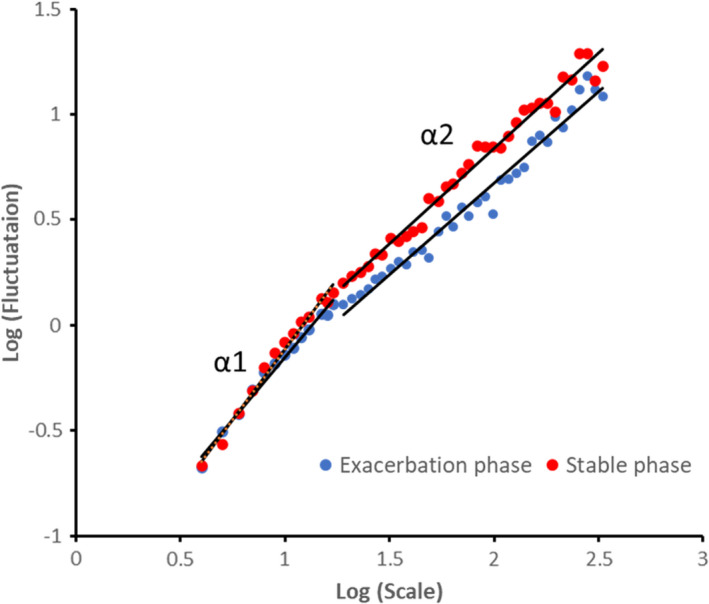
Two examples of DFA graphs on S_p_O_2_ variability data showing the linear trend when plotting scale and detrended fluctuations on a log‐log scale. This graph represents the stable phase (red dots) and the exacerbation phase (blue dots) of a participant with COPD. α1 and α2 are short‐term and long‐term scaling exponent respectively

**FIGURE B1:**
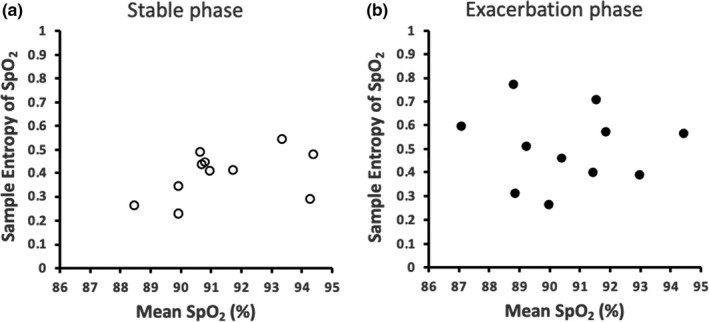
(a) Correlation between mean S_p_O_2_ and S_p_O_2_ Sample Entropy in individuals with COPD in Stable phase. (b) Correlation between mean S_p_O_2_ and S_p_O_2_ Sample Entropy in individuals with COPD in Exacerbation phase. There is no significant correlation between mean S_p_O_2_ and S_p_O_2_ entropy in individuals with COPD. This is unlike previous reports in healthy individuals where Entropy of S_p_O_2_ exhibits a significant inverse correlation with mean S_p_O_2_. For more information please see (Bhogal & Mani, 2017; Costello et al., 2020). Sample Entropy is calculated at scale 1 with m = 2 and r = 0.2

**FIGURE C1:**
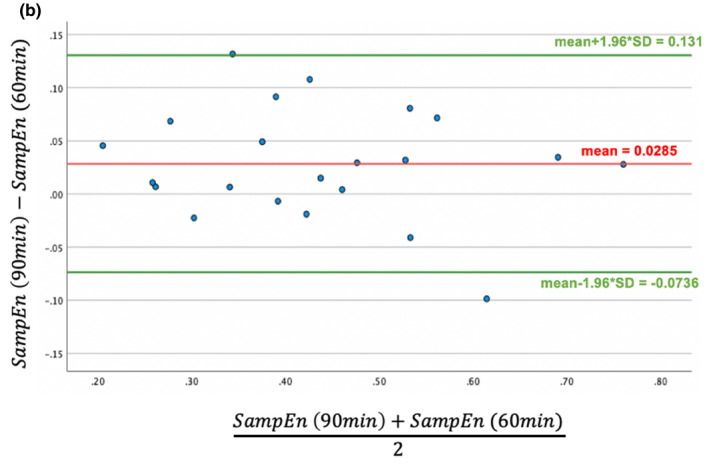
Bland‐Altman plot of sample entropy (SampEn) in 90 min vs. 60 min S_p_O_2_ signal duration

## Data Availability

The data that support the findings of this study are available on request from the corresponding author.
